# Giant Cell Arteritis in an Elderly Female With Pulmonary Tuberculosis

**DOI:** 10.7759/cureus.37805

**Published:** 2023-04-18

**Authors:** Ashwin Parchani, Ashish Baweja, Harnoor Singh, Yatharth Malik, Vinod Gupta, Minakshi Dhar, Monika Pathania

**Affiliations:** 1 Geriatric Medicine, All India Institute of Medical Sciences, Rishikesh, Rishikesh, IND; 2 Internal Medicine, Division of Rheumatology, All India Institute of Medical Sciences, Rishikesh, Rishikesh, IND

**Keywords:** polymyalgia rheumatica, large vessel vasculitis, atherosclerosis, tuberculosis, giant cell arteritis

## Abstract

Giant cell arteritis (GCA) is a type of systemic vasculitis that primarily affects people over the age of 50 and affects the medium to large arteries. GCA's clinical manifestations can be varied and non-specific, similar to those observed in atherosclerosis. Here, the authors present a case of an elderly woman with pulmonary tuberculosis with GCA masquerading as atherosclerosis.

## Introduction

Giant cell arteritis (GCA) is a systemic vasculitis affecting medium to large arteries, with a pooled incidence of 10 cases per 100,000 people over 50 years old [1]. It is a rare but serious condition that can cause significant morbidity and mortality if not diagnosed and treated promptly. The clinical presentation of GCA can be variable, with symptoms such as headaches, jaw claudication, and vision loss. However, these symptoms are usually less sensitive and specific, and may overlap with other common conditions such as atherosclerosis, making diagnosis challenging [2,3]. In this case report, we present a case of an elderly woman with pulmonary tuberculosis (TB) with GCA masquerading as atherosclerosis that highlights the importance of a thorough evaluation and a high index of suspicion for this rare but potentially critical illness.

## Case presentation

A 62-year-old lady, known case of type 2 diabetes mellitus, hypothyroidism and diagnosed microbiologically proven pulmonary TB four months ago, presented with right sided jaw pain on chewing food for the last three months. The pain was dull aching, insidious in onset and gradually progressive, without any history of toothache, ear discharge or oral ulcers. On further questioning, she reported episodic headache for the last 1.5 years, which were dull aching and throbbing in character initially confined to the right hemi-cranium (temporal) but gradually involved the whole cranium. There were no associated symptoms like nausea, vomiting, photophobia, phonophobia, dizziness or loss of consciousness but episodes of blurring of vision were reported. Over-the-counter medications provided relief. She also complained of bilateral shoulder pain for the past 1.5 years, insidious in onset, dull aching, associated with early morning stiffness, lasting for around one hour which eased with activity. This pain worsened over the last six months, hindering her ability to lift her arms overhead and perform household activities. There was no history of backpain, bluish discoloration of fingers, peripheral joint pain or swelling, chest pain, palpitations, cough, expectoration, syncopal attacks or fever. 

Initial imaging studies done previously at an outside hospital reported atheromatous changes in major arteries, based on ultrasonographic findings and magnetic resonance angiography of brain and neck. These vessel changes were attributed to atherosclerosis. Lab investigations revealed a normal lipid profile, normal Protein C and S activity. Her erythrocyte sedimentation rate (ESR) was raised significantly, with a value of 140 mm/hour. 

**Figure 1:**
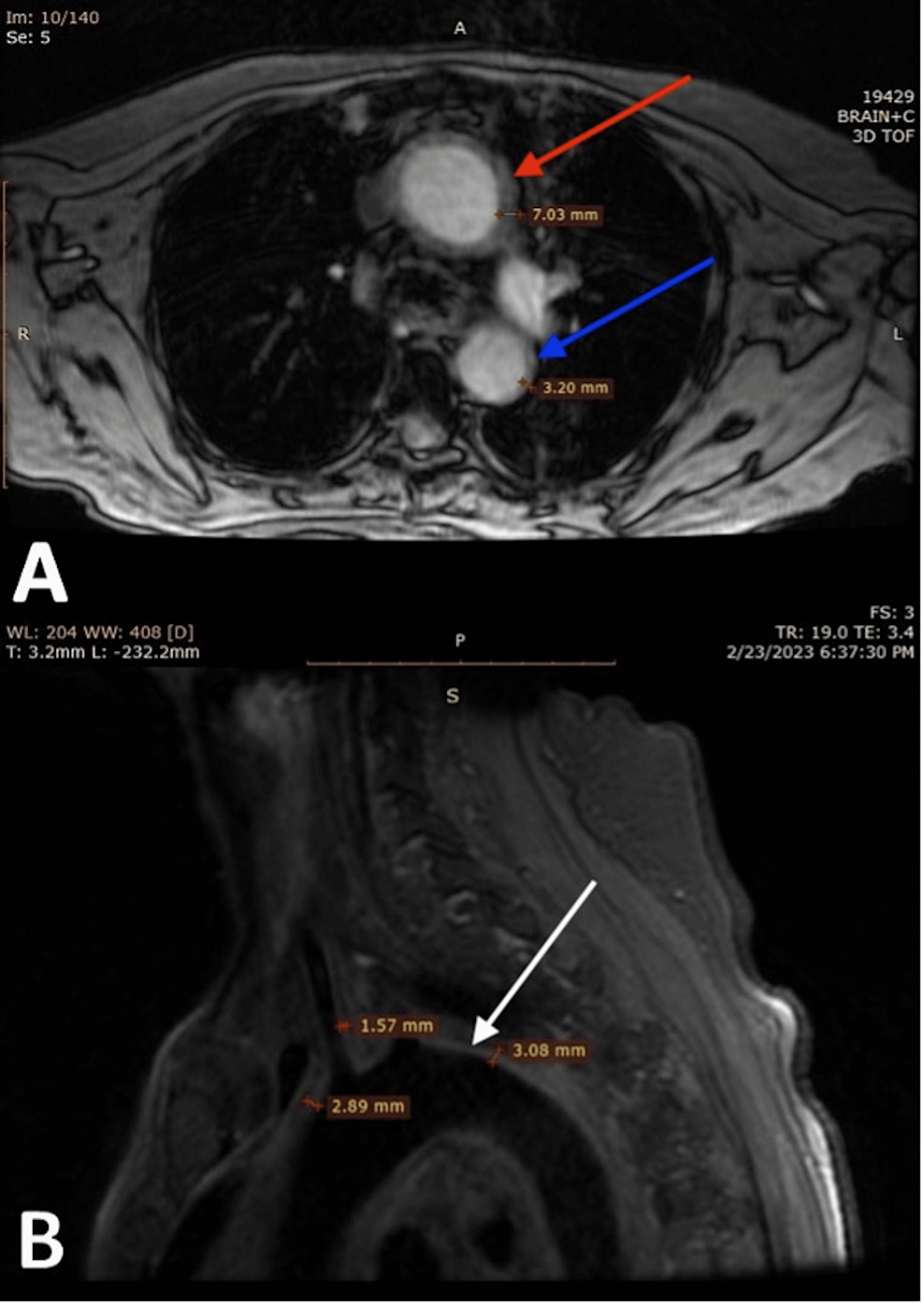
A) An axial MR-angiography showing circumferential mural thickening in ascending (red arrow) and descending (blue arrow) thoracic aorta. B) A sagittal section of MR-angiography showing mural thickening of arch of aorta (white arrow) and its branches.

**Figure 2:**
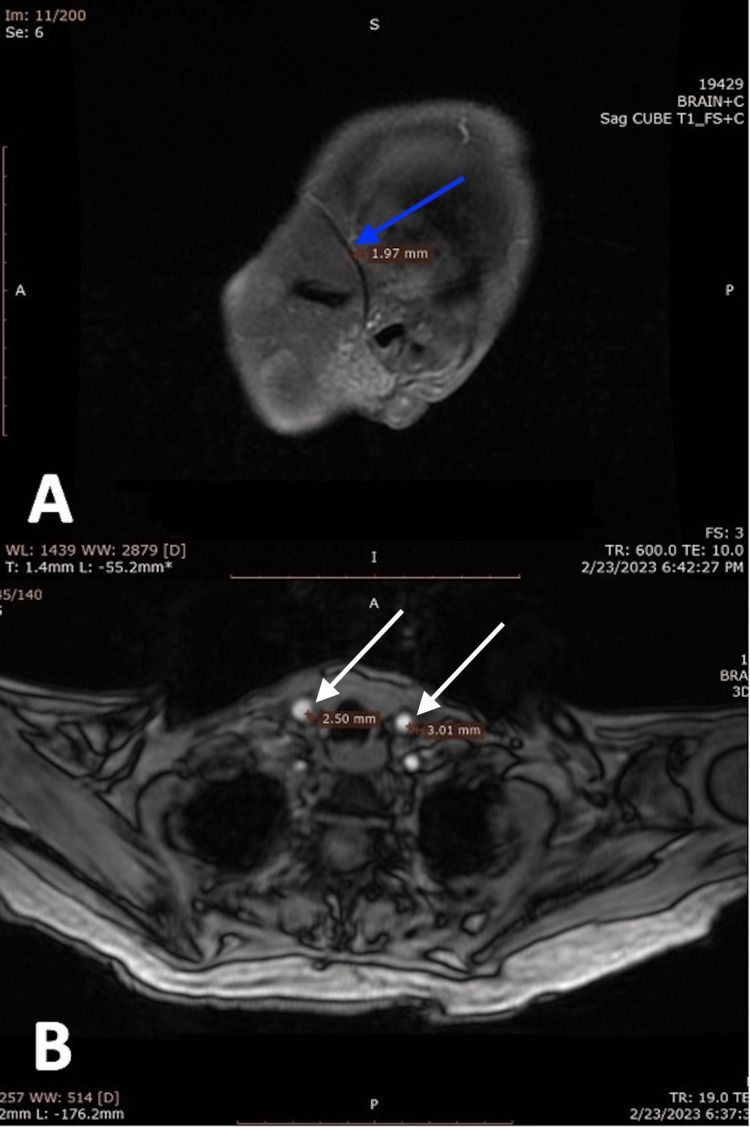
A) A sagittal section of MR-angiography showing thickening of temporal artery (blue arrow). B) An axial MR-angiography showing mural thickening in bilateral common carotid arteries (white arrows).

On physical examination, bilateral upper limbs were pulseless. There was a difference in blood pressures between upper limbs and lower limbs, with lower pressure in bilateral upper limbs (right arm: 84/50 mmHg, left arm: 78/48 mmHg, right leg: 160/46 mmHg, left leg: 162/48 mmHg). Fundoscopy showed moderate nonprogressive diabetic retinopathy changes in both eyes and mild temporal disc pallor in right eye. Thus, a contrast enhanced MRI for vessel wall imaging of brain and neck was done which revealed mural thickening and enhancement in bilateral common carotid arteries, aortic arch, arch vessels and bilateral temporal arteries, suggestive of large-vessel (LV) vasculitis (Figures 1, 2). A temporal artery ultrasound (TAUS) showed edematous thickening of both temporal arteries and axillary arteries. A Halo sign was also appreciated, with a total Halo score of 26 (Table 1). She was diagnosed with GCA based on clinical presentation and the 2022 American College of Rheumatology (ACR) classification criteria with LV-GCA pattern of involvement.

**Table 1:** Halo Score Calculation

	Artery	Halo thickness(mm)	Halo Grade	Halo Score
LEFT	Axillary	1.1	3	3*3 + 2 + 2 + 4*3 + 1 = 26
	Superficial Temporal	0.58	2	
	Frontal branch	No thickening seen	0	
	Parietal branch	0.44	2	
RIGHT	Axillary	2.1	4	
	Superficial Temporal	0.44	1	
	Frontal branch	No thickening seen	0	
	Parietal branch	No thickening seen	0	

## Discussion

Our case highlights the fact that GCA can be easily overlooked as it presents with clinical features similar to atherosclerosis. The diagnosis of GCA was made according to 2022 ACR Classification criteria which states that a patient is classified as having GCA with a cumulative score of ≥6 points. To make the diagnosis of GCA, we used TAUS which revealed presence of Halo sign. In this case, morning stiffness in shoulders, episodes of vision loss, jaw claudication, temporal headache, an ESR of 140 mm/hour supported by imaging showing Halo sign with a Halo score of 26, all led to the diagnosis of GCA with a score of 17 as per 2022 American College of Rheumatology/EULAR Classification Criteria criteria [4].

We employed the use of TAUS in our patient instead of a temporal artery biopsy. Numerous research conducted over the past 25 years compared the diagnostic accuracy of TAUS to that of temporal artery biopsy (TAB) and clinical criteria. When compared to the clinical diagnosis of GCA, the overall sensitivity and specificity of TAUS were 77% and 96%, with likelihood ratios of 19 and 0.2 for positive and negative US, respectively [5]. TAB has a lot of issues while evaluating possible GCA instances. In addition to being expensive and invasive, it has frequently been demonstrated to have a false-negative rate of up to 60%, most likely as a result of insufficient sample, skip lesions, and pre-operative steroid use [5,6]. A positive Halo sign on TAUS can be used to confirm a diagnosis of GCA in the presence of a high pre-test likelihood without the need for additional testing [6]. With the added advantages of better access and lower relative cost, it performs as well as MRI and PET/CT while being more sensitive and cost-effective than TAB. Another major advantage of TAUS over TAB is the time taken for reporting which can be up to 14 days for TAB. The use of TAUS may help in early detection of disease and prevention of permanent visual loss by prompt initiation of glucocorticoids [5,6].

In our case, the occurrence of jaw claudication started within two weeks of a diagnosis of pulmonary TB. Several theories have suggested a potential role of infections in the development of GCA [7,8]. The exact mechanisms of how TB infection could cause GCA are not yet fully understood. However, a similar process has been proposed for Takayasu arteritis where M. tuberculosis may trigger cross-reactivity against vascular peptides that mimic the antigens of M. tuberculosis [9]. While TB is classically associated with Takayasu arteritis, it is not commonly associated with GCA [10]. However, a recent study conducted in China found a higher incidence of TB in patients with GCA compared to the normal population [11]. Additionally, another study revealed a higher mortality rate in patients with TB and GCA compared to those with GCA alone [12]. This underscores the need for careful evaluation and appropriate management of patients with both TB and GCA, as these patients may have an increased risk of mortality when treated with corticosteroids [13]. Therefore, a thorough evaluation for TB should be performed in patients with suspected GCA, especially in areas with a high prevalence of TB. 

It's worth noting that atherosclerosis can mimic GCA symptoms, leading to a misdiagnosis. Therefore, when an elderly patient presents symptoms like vision loss, headaches, and claudication, GCA should be considered, and a comprehensive evaluation involving ESR and vessel wall imaging is necessary for an accurate diagnosis. The pathogenesis of atherosclerosis in the contemporary understanding involves an inflammatory process that can be accelerated by systemic inflammation [14]. Also, cardiovascular events have been demonstrated to be more prevalent in GCA patients, occurring more commonly within one year of diagnosis of GCA, indicating that vasculitis or inflammation-induced endothelial dysfunction and plaque instability may cause vascular ischemia [15]. Therefore, it is crucial to comprehend the intricate relationship between inflammation and atherosclerosis, particularly in patients with underlying inflammatory conditions like GCA.

## Conclusions

Our case highlights the potential for GCA to be misdiagnosed as atherosclerosis, emphasizing the need for thorough evaluation of elderly patients with symptoms like vision loss and headaches. The occurrence of GCA after TB raises the possibility of a link between the two conditions, and further research is needed to understand this relationship. Although there is no conclusive evidence of accelerated atherosclerosis in patients with GCA, cardiovascular events are more prevalent, indicating the importance of understanding the relationship between inflammation and atherosclerosis in patients with underlying inflammatory conditions.

## References

[1] Li KJ, Semenov D, Turk M, Pope J (2021). A meta-analysis of the epidemiology of giant cell arteritis across time and space. Arthritis Res Ther.

[2] Armona J, Rodríguez-Valverde V, González-Gay MA, Figueroa M, Fernández-Sueiro JL, Blanco R, Martínez-Taboada V (1995). [Giant cell arteritis. A study of 191 patients]. Med Clin (Barc).

[3] Goodman BW Jr, Shepard FA (1983). Jaw claudication. Its value as a diagnostic clue. Postgrad Med.

[4] Ponte C, Grayson PC, Robson JC (2022). 2022 American College of Rheumatology/EULAR classification criteria for giant cell arteritis. Ann Rheum Dis.

[5] Schmidt WA (2018). Ultrasound in the diagnosis and management of giant cell arteritis. Rheumatology (Oxford).

[6] Kirby C, Flood R, Mullan R, Murphy G, Kane D (2022). Evolution of ultrasound in giant cell arteritis. Front Med (Lausanne).

[7] Lyons HS, Quick V, Sinclair AJ, Nagaraju S, Mollan SP (2020). A new era for giant cell arteritis. Eye (Lond).

[8] Russo MG, Waxman J, Abdoh AA, Serebro LH (1995). Correlation between infection and the onset of the giant cell (temporal) arteritis syndrome. A trigger mechanism?. Arthritis Rheum.

[9] Castillo-Martínez D, Amezcua-Guerra LM (2012). Self-reactivity against stress-induced cell molecules: the missing link between Takayasu's arteritis and tuberculosis?. Med Hypotheses.

[10] van Timmeren MM, Heeringa P, Kallenberg CG (2014). Infectious triggers for vasculitis. Curr Opin Rheumatol.

[11] Zhang Y, Wang D, Yin Y, Wang Y, Fan H, Zhang W, Zeng X (2019). Tuberculosis infection in Chinese patients with giant cell arteritis. Sci Rep.

[12] Chazal T, Lhote R, Rey G, Haroche J, Eb M, Amoura Z, Cohen Aubart F (2018). Giant-cell arteritis-related mortality in France: a multiple-cause-of-death analysis. Autoimmun Rev.

[13] Thomas K, Vassilopoulos D (2017). Infections and vasculitis. Curr Opin Rheumatol.

[14] Zhu Y, Xian X, Wang Z (2018). Research progress on the relationship between atherosclerosis and inflammation. Biomolecules.

[15] Clifford AH, Cohen Tervaert JW (2021). Cardiovascular events and the role of accelerated atherosclerosis in systemic vasculitis. Atherosclerosis.

